# Receptor-interacting protein in malignant digestive neoplasms

**DOI:** 10.7150/jca.57076

**Published:** 2021-05-19

**Authors:** Lilong Zhang, Wenyi Guo, Jia Yu, Chunlei Li, Man Li, Dongqi Chai, Weixing Wang, Wenhong Deng

**Affiliations:** Department of General Surgery, Renmin Hospital of Wuhan University, Jiefang Road 238, Wuhan, Hubei 430060, China.

**Keywords:** Receptor Interacting Proteins, malignant digestive neoplasms, Therapeutics, Necroptosis

## Abstract

A deep and comprehensive understanding of factors that contribute to cancer initiation, progression, and evolution is of essential importance. Among them, the serine/threonine and tyrosine kinase-like kinases, also known as receptor interacting proteins (RIPs) or receptor interacting protein kinases (RIPKs), is emerging as important tumor-related proteins due to its complex regulation of cell survival, apoptosis, and necrosis. In this review, we mainly review the relevance of RIP to various malignant digestive neoplasms, including esophageal cancer, gastric cancer, colorectal cancer, hepatocellular carcinoma, gallbladder cancer, cholangiocarcinoma, and pancreatic cancer. Consecutive research on RIPs and its relationship with malignant digestive neoplasms is required, as it ultimately conduces to the etiology and treatment of cancer.

## Introduction

Receptor Interacting Proteins (RIPs), also known as receptor interacting protein kinases (RIPKs), are a seven-member family of serine/threonine and tyrosine kinase-like kinases, including RIP1-7, which acts as a critical sensor of intracellular and extracellular stimuli and plays an important role in cell death, immune response and inflammation 1. These proteins share a homologous serine-threonine kinase domain (KD), and RIP2 displays an additional tyrosine kinase activity 2. In addition to the homologous kinase domain, RIP1, the founding member of the family, has a C-terminal death domain (DD) which mediates death-receptor signaling, and a bridging intermediate domain (ID) which includes a RIP homotypic interaction motif (RHIM); RIP2 bears a C-terminal caspase activation and recruitment domain (CARD) and an ID without RHIM; RIP3 only contains a C-terminal RHIM domain alongside the N-terminal KD; RIP4 and RIP5 harbor an ID without RHIM and a C-terminal ankyrin repeat domains (ARD), while RIP6 and RIP7 are structurally less similar to other members. They incorporate many additional domains, such as ARD, lceucine-rich repeats domains (LRRD), Ras (GTPase) of complex proteins (Roc) and C-terminal of Roc (COR) domains, and WD40 repeats domains (WDRD) 2. For a detailed discussion of the biology of RIPs, we refer readers to a recent review 3. Here, we mainly review the relevance of RIP to various malignant digestive neoplasms. Consecutive research on RIP and its relationship with malignant digestive neoplasms is needed, as it ultimately conduces to the etiology and treatment of cancer.

## Role of RIPs in cell survival, apoptosis, and necrosis

As we all know, apoptosis, a programmed cell death mechanism, acts as a natural barrier to prevent the development of cancer. However, resistance and evasion to apoptosis are also deemed incontrovertible features of cancer 4, and apoptosis resistance usually also contributes to tumorigenesis and drug resistance, leading to chemotherapy failure 5. Thus, excepting overcoming resistance to apoptosis, the development of methods to induce non-apoptotic forms of programmed cell death is imperative and attractive as an alternative therapy for cancer. Recently, necroptosis, also known as programmed necrosis, was found as a novel and regulable form of cell death that has a morphological similarity to necrosis and a mechanistic similarity to apoptosis 6. As a combination of apoptosis and necrosis, necroptosis has been shown to have the following dual effects on cancer 7: on the one hand, the core mediators of this pathway alone or in combination have been thought to contribute to cancer metastasis and progression 8, 9; On the other hand, it was also reported to be a "fail-safe" mechanism that prevents tumor development when apoptosis is compromised 10, 11.

Currently, RIP1 is one of the most intensively investigated types, which is considered as a crucial molecule in the pathways in regulating cell survival, apoptosis, and necroptosis. RIP1 acts downstream of some receptors, such as tumor necrosis factor receptor (TNFR)1, Toll-like receptor (TLR)3, TLR4, and death receptors (DRs), of which TNFR1 signaling is the most classic pathway^12^-^15^. Following TNF activation of TNFR1, the latter undergoes trimerization that induces the formation of a receptor proximal membrane-signaling complex (Complex I) via sequential recruitment of RIP1, TNFR1-associated death domain protein (TRADD), TNF receptor-associated factor (TRAF) 2/5, cellular inhibitors of apoptosis (cIAP) 1/2, and Linear ubiquitin chain assembly complex (LUBAC) subunits (E3 ubiquitin ligase complex that includes HOIP, HOIL1, and SHARPIN and adds M1-linked linear polyubiquitin chains on RIP1 and other Complex I components), transforming growth factor (TGF) β-activated kinase 1 (TAK1)/TANK-binding kinase (TAB) 2/3, and nuclear factor kappa B (NF-κB) essential modulator (NEMO)/IκB kinase (IKK) 1/2 complexes 16-19. Within this complex, RIP1 is a key regulator of cell fate 20. Subsequently, several ubiquitination and phosphorylation events come up at Complex I that promote cell survival, including the TAK1/MK2(mitogen-activated protein kinase (MAPK)-activated protein kinase 2)-, IKK1/2- and TBK1-dependent phosphorylation of RIP1 (early cell death checkpoint) and the TAK1- and IKK-dependent activation of the NF-kB signaling pathway (late cell death checkpoint) 21-26. Nuclear translocation of NF-κB transcription factors regulates the expression of proinflammatory and prosurvival genes 27, including pro-survival factor and caspase-8 homolog cellular Fas-associated DD-like interleukin-1β converting enzyme inhibitory protein (c-FLIP) 28, the members of the inhibitor of apoptosis protein (IAP) family 29 and the anti-apoptotic Bcl-2 family 30. Furthermore, because of the quick internalization of ligand-bound TNFR, those proteins in complex I and their posttranslational modification are consequently changed 31. For instance, the second mitochondria-derived activator of caspases (Smac) mimetic-induced degradation of cIAPs causes the de-ubiquitination of RIP1 by the de-ubiquitinating enzyme de-ubiquitinating enzyme (CYLD) and/or A20 32-34. Consequently, complex I converts into a cytoplasmic death-inducing signaling complex (Complex II), including FADD, TRADD, caspase-8, and RIP1, which is also referred to as “Ripoptosome”, is formed, inducing caspase-8 activation and apoptosis 35, 36. When the activity of caspase-8 is inhibited by pharmaceutical or genetic intervention, RIP1 associates with RIP3 via their RHIM domains to shape a crucial GLU protein complex (necrosome). This can cause activation and autophosphorylation of RIP3 that in turn recruits the pseudo-kinase mixed lineage kinase like (MLKL). MLKL phosphorylation induces its translocation to the plasma membrane and forms pores that result in necroptotic cell death 15, 37, 38.

## RIPs and esophageal cancer

Esophageal cancer (EC) is a serious malignant tumor of the upper gastrointestinal tract with high mortality and poor prognosis 39. Recently, RIP3 expression level in esophageal squamous cell carcinoma (ESCC) has been described. The cytoplasmic expression of RIP3 mRNA and protein was significantly down-regulated in ESCC tumor tissues. And the expression of RIP3 was more inadequate in the more advanced and invasive tumors 40. Besides, RIP3 deficiency was associated with poor prognosis and cisplatin resistance in ESCC patients who had received cisplatin chemotherapy, and re-expression of kinase-dead RIP3 can restore cisplatin sensitivity 40. Further functional analyses found that RIP3 depletion stimulated the DNA repair pathway to potentiate cisplatin chemoresistance. Specifically, the deficient expression of RIP3 upregulated POLD1 and FOS Like Antigen 1 (FOSL1) via activation of the ERK phosphorylation and HSP90/CDC37 complex in ESCC cell lines 40. POLD1 is the DNA polymerase delta catalytic subunit and involves in nuclear excision repair 41, 42. Knockdown of POLD1 reduced repair activity and sensitized cells to reagents that cause DNA damage. Also, FOSL1 was upregulated in ESCC tumors, and the down-regulation of FOSL1 expression can inhibit cell proliferation and metastasis 43. Thus, RIP3 may be a useful marker for predicting chemotherapy sensitivity and prognosis of EC patients. And the RIP3 levels in tumor tissues may guide the chemotherapeutic regimen in the clinic.

## RIPs and gastric cancer

Gastric cancer (GC), a highly fatal disease, is a common malignancy type of gastrointestinal tract worldwide, with earlier stages being asymptomatic and difficult to detect, by the time of diagnosis it reaches the advanced stages 44. A recent study reported 45 that RIP1 was strongly associated with the occurrence and development of GC. The high expression of RIP1 was detected in GC tissues, while low expression of RIP1 was found in the normal gastric tissues 45. Importantly, immunohistochemistry staining revealed the RIP1 immunoreactivity was positive at the site of invasion with little or no immunoreactivity in the interstitial substance. And the RIP1 expression was significantly positively related to the clinical stage and lymph node metastasis of GC patients, which revealed that RIP1 may promote growth and invasion of GC 45. Further analysis showed the GC patients with low expression of RIP1 had a better prognosis than the patients with high expression of RIP1. Mechanistic studies found that the RIP1-NF-κB/AP-1-VEGF-C signaling pathways play a vital role in regulating the biological functions of human GC cell lines (HGC and AGS) 45. Among them, the VEGF-C is an essential factor for lymph node metastasis in the GC 46. The nude mice model with subcutaneous xenograft tumors further verified the above findings. Thus, RIP1 promotes the growth and invasion of GC *in vitro* and *in vivo*, targeting the overexpressed RIP may be used in the treatment of GC patients. Interestingly, the study also found that the expression of RIP1 was significantly higher in GC patients with Helicobacter pylori infection. The reason may be that Helicobacter pylori infection causes inflammation, which increases the RIP1 expression 47.

Also, studies have also found that RIPs may be involved in anti-gastric cancer treatment. Celastrol, isolated from the root of Thunder of God Vine, has anti-inflammatory and anti-cancer activity. Guo *et al*. confirmed 48 that celastrol can activate the RIP1/RIP3/MLKL pathway by down-regulating biglycan to result in the necroptosis of HGC-27 and AGS cell. Previous research reported that biglycan expression was upregulated in GC tissues to enhance GC invasion 49. Furthermore, RIP3 knockdown, apoptosis inhibitor (Z-VAD-fmk) or necrostatin-1 (Nec-1), the first well-established necroptosis inhibitor that exclusively suppressed RIP1 activity 50, all can in part rescue celastrol-induced GC cell death. Vetrivel et al. 51 also came up with similar results through *in vitro*, vivo experiments, and silico molecular docking and simulation studies. They found that prunetin induced necroptosis-mediated human AGS cell death via activating the RIP3, leading to the phosphorylation of MLKL. As the third-generation of platinum anti-cancer drugs, oxaliplatin is widely used in various cancers, which works by binding to DNA to produce oxaliplatin-DNA conjugates. However, the mechanism by which the DNA conjugate kills cells is not fully clear. Recently, Wu *et al*. 52 demonstrated that oxaliplatin killed human GC cell SGC-7901 primarily by necroptosis relying on RIP1, and Nec-1 significantly inhibited oxaliplatin-induced cell death.

## RIPs and colorectal cancer

Colorectal cancer (CRC) is the third most common human cancer and the second leading cause of cancer death worldwide 53. It is considered a multifactorial disease, whose etiology is relevant to genetic factors, environmental exposure, and inflammatory conditions of the intestinal tract 54. However, the underlying mechanisms of CRC carcinogenesis remain unclear. Recently, some studies suggested that RIP1 may be involved in CRC colorectal cancer oncogenesis. For instance, Zeng *et al*. 55 discovered that, compared to normal para carcinoma tissue, the RIP1 protein and mRNA expression levels were upregulated significantly in human CRC tissues, and RIP1 protein levels were positively related to the stage of cancer progression and 3 years mortality rate. Further analysis showed that the mitochondrial Ca^2+^ uniporter (MCU), an evolutionarily conserved Ca^2+^ channel, was a crucial interacting directly downstream component of RIP1 in promoting cellular proliferation in the colon cancer cell line of HT29 by increasing energy metabolism and mitochondrial Ca^2+^ uptake. Besides, the study also found that knockdown of RIP1 or MCU could significantly impede cancer growth in a nude mouse tumor inoculation model. Thus, targeting RIP1 and MCU may be a new strategy for the treatment and prevention of CRC. Moriwaki *et al*. 56 drew the opposite conclusion. They found that, compared with adjacent normal colon tissues, the expression of RIP1 and RIP3 was remarkably decreased in human colon cancer tissue. As we know, the expression of tumor suppressor genes is often silenced in cancer tissues by epigenetic DNA modifications such as DNA methylation and histone deacetylation 57. However, Moriwaki* et al* found that RIP1 and RIP3 expression were suppressed by hypoxia, a hallmark of solid tumor, but not by epigenetic DNA modification. Hypoxia has been implicated to promote cancer progression by activating adaptive transcriptional programs that promote cell survival and angiogenesis 58.

Other studies also found that the expression of RIP3 was significantly decreased in human CRC tissues compared with adjacent normal tissues 11, 40, 59. The down-regulation expression of RIP3 was associated with some disadvantageous clinicopathologic parameters such as T stage, M stage, and AJCC stage 11, and damaged the colon cancer cells' response to necroptosis triggers 56. Overexpression of RIP3 can suppress proliferation, migration, and invasion of CRC cell lines *in vitro* 11. COX risk model analysis indicates that the expression level of RIP3 was an independent prognostic factor for disease-free survival and overall survival in CRC patients, and RIP3 negative patients had a remarkably lower survival rate than patients with RIP3 positive staining 11. The downregulation of RIP3 in tumor-infiltrating myeloid-derived suppressor cells (MDSCs) potentiates NF-kB activation and COX-2-derived prostaglandin E2 (PGE2) production. PGE2, in turn, further decreases RIP3 expression level and promotes the immunosuppressive activity of MDSCs and colorectal carcinogenesis 60. Bozec *et al*. 59 revealed that mice with RIP3-deficient were highly susceptible to colitis-associated CRC and displayed greater production of tumor-promoting factors and pro-inflammatory mediators. The tumorigenesis of RIP3 deficiency results from uncontrolled activation of AKT, STAT3, NF-κB, and Wnt-β-linked protein signaling pathways that promote the proliferate abnormally of intestinal epithelial cells (IECs) and CRC. Thus, RIP3 may play a key anti-inflammatory and anti-tumoral functions in the intestine. Besides, Conev *et al*. 61 revealed that metastatic colon cancer patients with a high level of RIP3 expression were related to longer overall survival and progression free survival, and lower risk of disease progression. This study also found that patients with high expression of RIP3 had a significantly higher response rate to 5-fluorouracil based chemotherapy regimens, which indicated RIP3 expression levels might be a potential predictive marker for response rate. However, at the level of RIP3 expression, Liu *et al*. and He *et al*. came to the opposite conclusion. They revealed that RIP3 expression levels were upregulated in human and mouse colonic cancers and mouse colitis-associated cancer (CAC) 62, 63, and RIP3 deficiency decreased significantly colitis-associated tumorigenesis. Mechanistic studies revealed that, on the one hand, RIP3 enhanced the proliferation of premalignant intestinal epithelial cells to facilitate the progression of CAC via activating JNK signaling; on the other hand, RIP3 promoted the myeloid cell-induced adaptive immune suppression to contribute to the CAC progression by CXCL1 signaling to increase the number of myeloid-derived suppressor cells and M2c-like macrophage tumor-associated macrophages while decreasing T-cell accumulation, infiltration and activation. Therefore, activation and expansion of T cells by the impediment of RIP3 signaling are a promising way to raise T cell activity and strengthen the effectiveness of cancer immunotherapy 62. The different expression patterns of RIP1 and RIP3 in colon cancer may be a consequence of its pleiotropic functions in multiple signaling pathways, and the expression of RIP1 and RIP3 in CRC patients deserves further study.

Apart from interfering with the development of CAC, RIP also affects anti-colorectal cancer treatment. Currently, 5-Fluorouracil (5-FU), irinotecan (the active metabolite of which is SN38), and oxaliplatin are the major chemotherapeutic agents in the treatment of CRC. However, CRC patients have a low response rate to these agents, and even with positive treatments, the 5-year survival rate of patients with advanced diseases is < 10%. Thus, increasing these agent sensitivities in resistant tumors remains an urgent challenge in the chemotherapy of CRC. Recently, some scholars have found that RIP1 can affect the sensitivity of anti-colorectal drugs. Metzig *et al*. 64 revealed that 5-FU and pan-caspase inhibitor Z-VAD-induced necroptosis in CRC cells depends on RIP1 and RIP3, and knockdown of RIP1 and RIP3 significantly rescued cells from 5-FU plus Z-VAD-induced necroptosis. Other scholars have found that the downregulation of RIP1 can increase resistance to topoisomerase inhibitor 7-Ethyl-10-hydroxycamptothecin (SN38) in the colon cancer cell line of HT29 and HCT116 under hypoxic conditions or normoxic, and SN38 alone and in combination with TNF declined the expression RIP1 in the treatment for HT29 cells. RIP1 knockdown HT29-derived tumor xenograft models have demonstrated that RIP1 was required for tumor growth and the tumors that lack RIP1 grow more slowly. Further, the therapeutic benefit of irinotecan was apparent in the control mice, while the tumor mice of RIP1 knockdown had no obvious response to irinotecan 65.

Resibufogenin, derived from toad venom, triggers necroptosis to inhibit growth and metastasis of colorectal cancer via upregulating RIP3 and MLKL. Besides, resibufogenin also activated the expression of glycogen phosphorylase (PYGL), glutamine synthetase (GLUD1), and glutamate dehydrogenase (GLUL) in a RIP3-dependent manner to promote a considerable metabolic burst 66. This in turn propagates the overgeneration of ROS, thereby favoring mitochondrial dysfunction and necroptosis 67, 68. Mishra *et al*. 69 discovered that the overexpression of GLTP, encoding a 24 kD amphitropic lipid transfer protein, can induce cell death by necroptosis in HT-29 colon cancer cells as explained by RIP3-induced the phosphorylation of mixed lineage kinase domain-like protein (pMLKL), elevated intracellular Ca^2+^ levels and the permeabilization of the plasma membrane by pMLKL oligomerization, but not in HCT-116 or normal colonic cells. To further determine the RIP3 dependence of GLTP-induced HT-29 cell death, the author depleted RIP3 or MLKL, and found the above-mentioned conditions invalidated necroptosis induced by the overexpression of GLTP. Some scholars have also pointed out that 2-methoxy-6-acetyl-7-methyljuglone (MAM) can promote the death of HCT116 and HT29 colon cancer cells, and RIP1/RIP3 complex-induced cytosolic calcium accumulation is a key mediator in MAM-induced necroptosis in human colon cancer cells via mitochondrial ROS production and sustained JNK activation 70.

In addition to necroptosis, RIP3 is also involved in other non-apoptotic programmed cell death pathways that lead to CRC cell death, such as autophagy. Hou *et al* 71 first discovered that chloroquine (CQ) upregulated the expression of RIP3 in CT26 cancer cells, and RIP3 can participate in CQ-related autophagy. The combination of mRIP3 and CQ had a significant antitumor effect *in vitro* and *in vivo* experiments. Furthermore, this combination can enhance lysosomal membrane permeabilization via increasing autophagic flux and inducing RIP3-dependent necroptosis, but apoptosis was not increased.

## RIPs and hepatocellular carcinoma

Hepatocellular carcinoma (HCC) accounts for the fourth leading cause of cancer death globally and has highly invasive and aggressive behavior with 5-year survival rates of 3%-11% 72, 73. Recently, some scholars have found that patients with higher expression of RIP1 in HCC tissues suffered from poor post-surgical survival. The RIP1 expression was significantly increased in HCC tissues than in adjacent normal liver tissues from 81.9% of HCC patients. And RIP1 overexpression was found positively related to advanced TNM staging, intrahepatic metastases, portal vein invasion, and HBV infection 74. Mechanistic studies revealed that RIP1 exerted its function on HCC progression through the activation of the AKT/Bcl-2/BAX signaling pathway 74. However, Schneider *et al*. 75 reached the opposite conclusion. They revealed that low expression of RIP1 and TNF receptor-associated factor 2 (TRAF2) in HCC was related to poor prognosis. Further analysis showed RIP1 deficiency in liver parenchymal cells (LPC) enhanced the degradation of TNF-induced TRAF2, causing liver injury. And the loss of both RIP1 and TRAF2 in LPC promoted the spontaneous development of HCC, which suggests that RIP1 collaborates with TRAF2 to suppresses hepatocarcinogenesis 75. Besides, Van *et al*. investigated the role of RIP1 kinase-dependent and -independent functions in hepatic homeostasis, hepatocyte death, liver injury, and hepatocarcinogenesis. This work has demonstrated that kinase-independent RIP1 functions (scaffolding functions) with NF-κB to prevent hepatocyte apoptosis, chronic liver disease, and cancer under steady-state conditions. However, when NEMO is absent, RIP1 kinase activity drives hepatocyte apoptosis, chronic liver disease, and HCC development 76. Recently, RIP6 has also been found to inhibit the growth of hepatocellular carcinoma cells. The work revealed that, compared to adjacent normal tissues, RIP6 was significantly down-regulated in human HCC tissue. And, the low expression of RIP6 was positively associated with tumor size in HCC patients. Further studies found that RIP6 induced HCC cell apoptosis to suppress cancer progress, and inhibited HCC cell proliferation to further prevent cancer growth 77.

The IKK complex consists of the catalytic subunits IKKα and IKKβ, and the regulatory subunit NEMO. IKKα and IKKβ, known function in NF-κB activation, also directly phosphorylate RIP1 at different regions of the protein to regulate cell viability 78. Loss of this IKKα/β-dependent RIP1-phosphorylation in LPC impedes compensatory hepatocytes and intrahepatic biliary cell s proliferation, thus inhibiting the development of HCC but inducing biliary cell paucity and lethal cholestasis 79. Kondylis *et al*. 80 discovered that mice lacking NEMO in LPC spontaneously develop steatohepatitis and HCC. RIP1 kinase activity-induced hepatocyte apoptosis can drive HCC in NEMO^LPC-KO^ mice. And NEMO prevents hepatocarcinogenesis by inhibiting RIP1 kinase activity-mediated hepatocyte apoptosis via NF-kB-dependent and NF-kB -independent functions 80. Interestingly, Aigelsreiter *et al*. 81 reported that loss of NEMO immunoreactivity in a considerable percentage of human HCC tissues, which related to a poor 5-year overall survival. Thus, the inhibition of RIP1 could provide a likely well-tolerated and effective therapy choice for this subset of patients. Besides, Clinical practice has shown that inhibition of RIP1 promotes the anti-tumor effect of pirarubicin by the RIP1-AKT-P21-dependent pathway to overcome chemoresistance in HCC 82.

HepG2/DDP cells, a human liver cancer cell line HepG2 cisplatin-resistant counterpart, are resistant to cisplatin-induced apoptosis due to downregulation of pro-apoptotic genes (Bad and Bax) and upregulation of anti-apoptotic genes (Bcl-XL and Bcl-2) 83. Zhang *et al*. 84 discovered that ectopic expression of RIP3 prompted cisplatin-induced HepG2/DDP cell death. And this type of cell death was necroptosis and relied on the RIP1-RIP3-MLKL signaling pathway owing to inhibition of MLKL activity by necrosulfonamide (NSA) or knockdown of RIP1 significantly reduced cisplatin-induced cell death. The author also found that the high level of RIP3 expression was closely related to better response rates to cisplatin clinical treatment in HCC patients. However, the expression of RIP3 was usually inhibited in liver tumor cells due to genomic methylation. Thus, RIP3-dependent activation of necroptosis was also mostly repressed during chemotherapy. A previous study reported that hypomethylating agents could restore RIP3 expression level in some cancer cells, enhancing their sensitivity to chemotherapeutics-induced cell necroptosis 85. Therefore, the combined use of hypomethylating agents and chemotherapeutics may be a better choice for the treatment of HCC patients. The result of Li *et al*. 86 and Sun *et al*. 40 is consistent with the above findings. They also confirmed that the expression of RIP3 is declined in HCC patients, which correlates with myeloid-derived suppressor cell (MDSC) infiltration and poor prognosis. RIP3 deficiency facilitates CXCL1/CXCR2-induced MDSC chemotaxis to promote immune escape and HCC progression. And MDSC is regarded as a major suppressor of T-cell cytotoxicity and a major mechanism adopted by cancers to escape immune surveillance 87, 88. Other scholars also have reported that RIP3 hinders inflammatory hepatocarcinogenesis but induces cholestasis via controlling caspase-8-and Jun-(N)-terminal kinase (JNK)-dependent compensatory cell proliferation 89.

Liver oval cells (OCs) have highly proliferative activity, which when disrupted may promote HCC 90. Interestingly, WÓJCIK *et al*. 91 indicated that, compared with the non-neoplastic OCs, the OCs of neoplastic livers possess high proliferative activity with apoptosis suppression, and their necroptosis potential was preserved, which was reflected by the pronounced RIP3 expression and unchanged RIP1 expression. However, the authors also suggest that although this response strengthens necroptotic cell death, it is not sufficient to disrupt the highly proliferative activity of neoplastic OCs.

## RIPs and gallbladder cancer

Gallbladder cancer (CA) is a very rare, highly-lethal, highly invasive and aggressive, and carries a dismal prognosis disease. The 5-year survival rate for all stages of CA patients is about 5% 92, 93. The high expression of RIP1 was found in the CA tissues, but low expression of RIP1 was detected in the normal gallbladder tissues. The patients with IV~V clinical stage, lymph node metastasis, and gallstones had higher levels of RIP1 expression, which indicated that RIP1 may promote tumor invasion and growth 94. Notably, gallstones can cause inflammation of the gallbladder, which can increase RIP1 expression 47, while the direct relationship between gallstones and CA has long been established 95. Intriguingly the subcutaneous xenograft tumors model confirmed that RIP1 promoted tumor growth in nude mice 94. And silencing of RIP1 in gallbladder cell lines NOZ significantly repressed proliferation and invasiveness by downgrading the RIP1-NF-κB(p65)/C-jun(AP-1)-VEGF-C pathways 94. Thus, targeting RIP1 for CA may be a promising option.

## RIPs and cholangiocarcinoma

Cholangiocarcinoma (CCA) is an aggressive malignancy originating from the epithelium of extrahepatic and intrahepatic bile ducts, leading to high mortality and recurrence/metastasis rates and subsequently poor clinical outcomes, with a relatively low 5-year survival rate (5-10%) 96, 97. Recently, Sacchi *et al*. 98 confirmed that RIP played an important role in the growth and treatment of CCA. The RIP3, RIP1, and pMLKL were highly expressed in the intrahepatic CCA patients. Importantly, these necroptosis-associated proteins were found to be negative correlated with the presence of perineural invasion and nodal metastasis. And the patients with higher RIP3 and RIP1 expression have a significantly longer overall survival 98. Another study analyzing RNA sequencing data from The Cancer Genome Atlas (TCGA) database found that both RIP3 and MLKL mRNA were upregulated in CCA tissues compared to in normal bile ducts, while RIP1 mRNA was similarly expressed between the two groups 99. However, A clinical study by Xu *et al*. 100 revealed that, compared to adjacent normal tissues, RIP3 was lower but still moderately expressed in most CCA tissue. Therefore, the relationship between RIP1, RIP3 and the development of CCA needs further study.

Besides, the role of RIPs in the treatment of CCA has also attracted the attention of relevant scholars. They revealed that the Smac mimetic, an inhibitor of apoptosis protein (IAP) antagonist, sensitized CCA cells to low-dose standard chemotherapy, gemcitabine, and induced necroptosis in a RIP1/RIP3/MLKL-dependent manner based on caspase inhibition, but not in non-tumor cholangiocytes 99. Xu *et al*. 100 showed that matrine, an alkaloid derived from traditional Chinese medicine Sophora flavescens, can induce necroptosis in CCA cell lines. CCA cells with matrine therapy displayed typical necrosis-like instead of apoptotic morphology changes, and these effects were considerably weakened by Nec-1, but not apoptosis inhibitor z-VAD-fmk. Further analysis showed matrine could enhance RIP3 expression level and the following RIP3/MLKL/ROS signaling pathway, which may contribute to the necroptosis process. And necroptosis was switched to apoptosis after knocking down RIP3 100. The findings have pivotal suggestions for designing a new necroptosis-based therapeutic ideal to overcome CCA.

## RIPs and pancreatic cancer

Pancreatic cancer (PC) is one of the deadliest solid cancers, with a 5-year survival rate of only about 6%, and patients with PC have the lowest survival rate of all patients with cancer 101, 102. Recently, Seifert *et al*. reported that RIP1, RIP3, FADD, caspase 8, and MLKL were highly expressed in pancreatic ductal adenocarcinoma (PDA) 103. The deletion of RIP3 or inhibition of RIP1 prevented the progression of PDA in mice *in vivo* and was related to the development of T cell infiltrate and a highly immunogenic myeloid. Mechanistic studies revealed that the immunosuppressive tumor microenvironment related to intact RIP1/RIP3 signal was partially dependent on the expression of necroptosis-induced chemokine attractant CXCL1, and blockade of the CXCL1 can protect against PDA 103. Besides, cytoplasmic SAP130, a subunit of the histone deacetylase complex, was upregulated in a RIP1/RIP3-dependent manner in PDA. Mincle, SAP130 cognate receptor which can promote sterile inflammation by ligating SAP130 104, 105, was upregulated in PDA tumor-infiltrating myeloid cells. Mincle deletion can protect against oncogenesis 103. Thus, the RIP1 and RIP3 promote PC progression through mincle and CXCL1-induced immune suppression. Previous studies have found that PC patients with preoperative serum CEA+/CA125+/CA19-9 ≥ 1000 U/ml usually had poor surgical outcomes and were more likely to develop distant metastases after radical surgery 106. A recent study 107 reported that RIP4 was one of the most key genes upregulated in this subgroup of patients. Further analysis showed the overexpression of RIP4 significantly stimulated PC cell invasion and migration by the proteasome-mediated phosphatidylethanolamine binding protein 1 (PEBP1) degradation-induced activation of the RAF1/MEK/ERK pathway. Among them, PEBP1, as a physiological endogenous inhibitor of the RAF1/MEK/ERK pathway, can impede the proliferation, migration, and invasion of PC cells 108.

The evasion of apoptosis is a critical mechanism of PC treatment resistance and leads to a poor prognosis 109. Some studies have found that necroptosis is a novel and alternative treatment strategy to induce programmed cell death in PC cells with apoptosis-resistant. For instance, Hannes *et al*. 110 revealed that the Smac mimetic BV6 can switch TNF-α into a death signal to induce necroptosis in PC cells when caspase activation was blocked. BV6 promoted the production of TNF-α and the formation of RIP1/RIP3-containing necrosome complex in PC cells. The pharmacological inhibition of RIP1, RIP3, or MLKL remarkably reduced BV6-induced cell death. Also, Doxorubicin sensitized human PC and CRC cells to Smac mimetic through synergistically activating CYLD/RIP1/FADD/caspase-8-dependent apoptosis. And RIP1 is critical for the synergistic induction of apoptosis by Smac mimetic and doxorubicin, while it is irrelevant for cell death caused by doxorubicin alone 111. Chen *et al*. 112 found that Shikonin, a naphthoquinone isolated from the Chinese medicinal herb Lithospermum erythrorhizon, can up-regulate RIP1 and RIP3 expression to induce apoptosis and necroptosis in different PC cells, and also strengthen the anti-tumor activity of gemcitabine. Other scholars have proposed that gemcitabine treatment can induce the components of the necrosome by increasing the expression level of RIP1 and RIP3 in PDA *in vivo* and *in vitro* 103. The combinatorial treatment of gemcitabine and CD95 ligand has also been demonstrated to induce apoptotic and RIP1-mediated necroptotic cell deaths in PC cells, and gemcitabine significantly switched CD95L-induced cell death into necroptosis 113.

In addition to chemotherapy and natural compounds that induce necroptosis in PC, metal nanoparticles also can induce necroptotic cell death. Silver nanoparticles, as metal nanoparticles, have received increasing attention and were regarded as a promising anticancer agent due to their unique functions to easily cross biological barriers and penetrate cell membranes to induce apoptosis in cancer cells 114-116. Based on a study performed by Zielinska *et al*., in human pancreatic cancer cells (PANC-1 cells), silver nanoparticles can promote both necroptosis and apoptosis and considerably increase the levels of proteins related to necroptosis and autophagy, including RIP1, RIP3, MLKL, and LC3-II. The author also pointed out that silver nanoparticles markedly inhibited the proliferation and reduced the viability of PANC-1 cells. And, PC cells were more remarkably sensitive than nontumor PC to silver nanoparticles-induced cytotoxicity 114. Thus, silver nanoparticles-induced cell death may provide a new therapeutic strategy to overcome chemoresistance in PC cancer.

## Conclusion

Overall, significant progress has been made in the field of RIPs biology, and there is a strong link between RIPs and the development and treatment of gastrointestinal malignant cancers. Continued in-depth research in this area will contribute to enhance our understanding of cancer control. However, as shown in Table 1, the study of RIPs in colorectal cancer, hepatocellular carcinoma, and cholangiocarcinoma remains controversial. In addition, current studies have focused on RIP1 and RIP3 and gastrointestinal malignancies, while no studies have been conducted on RIP2, PIR5, or RIP7. Recent studies have found that RIP2 promotes cell survival by activating NF-ĸB in triple-negative breast cancer cells 117. Thus, whether RIP2, RIP5, and RIP7 are involved in the development of malignant tumors in the gastrointestinal tract deserves further investigation. Finally, RIPs play an important role in a variety of antitumor therapies (Table 2), either by inducing necroptosis to bypass acquired or intrinsic apoptotic resistance or by increasing sensitivity to antitumor drugs. Thus, RIPs kinases are regarded as potential and promising therapeutic targets for cancer.

## Figures and Tables

**Table 1:** Expression of RIPs in malignant digestive neoplasms and its influence on cancer prognosis

Cancer Type	Expression of RIPs	The influence on prognosis	Reference
Esophageal cancer	Decrease PIR3 expression	Poor prognosis; cisplatin resistance; enhanced tumorigenesis	40
Gastric cancer	Increase RIP1 expression	Poor prognosis; enhanced tumorigenesis	45
Colorectal cancer	Increase RIP1 expression	Poor prognosis; enhanced tumorigenesis	55
Colon cancer	Decrease RIP1/RIP3 expression	Promoted oncogenesis	56
Colorectal cancer	Decrease RIP3 expression	Poor prognosis; enhanced tumorigenesis	11, 40, 59
Colorectal cancer	Increase RIP3 expression	Promoted oncogenesis and tumorigenesis	62, 63
Hepatocellular carcinoma	Increase RIP3 expression	Poor prognosis; enhanced tumorigenesis	74
Hepatocellular carcinoma	Decrease RIP6 expression	Inhibited tumorigenesis	77
Hepatocellular carcinoma	Decrease RIP3 expression	Poor prognosis	40, 86
Gallbladder cancer	Increase RIP1 expression	Enhanced tumorigenesis	94
Intrahepatic cholangiocarcinoma	increase RIP1/RIP3 expression	Good prognosis; inhibited tumorigenesis	98
Cholangiocarcinoma	Increase RIP3 expression; while RIP1 remains unchanged		99
Cholangiocarcinoma	Decrease RIP3 expression		100
Pancreatic cancer	Increase RIP1/RIP3 expression	Promoted oncogenesis	103
Pancreatic cancer	Increase RIP4 expression	Enhanced oncogenesis and tumorigenesis	107

**Table 2:** Compounds that induce cell death in cancer therapy

Compounds and Agents	Antitumor Mechanism	Cancer Type	Reference
Celastrol	RIP1/RIP3/MLKL pathway dependent	Gastric cancer	48
Prunetin	RIP3 dependent	Gastric cancer	51
Oxaliplatin	RIP1 dependent	Gastric cancer	52
Resibufogenin	Upregulating RIP3 and MLKL	Colorectal cancer	66
GLTP	Upregulating RIP3 and MLKL	Colorectal cancer	69
2-methoxy-6-acetyl-7-methyljuglone	Relying on RIP1/RIP3 complex	Colorectal cancer	70
Chloroquine	Upregulating RIP3	Colorectal cancer	71
Smac mimetic	RIP1/RIP3/MLKL-dependent manner	Cholangiocarcinoma	99
Matrine	Relying on RIP3/MLKL/ROS signaling pathway	Cholangiocarcinoma	100
Smac mimetic BV6	Formatting RIP1/RIP3-containing necrosome complex	Pancreatic cancer	110
Shikonin	Upregulating RIP1 and RIP3	Pancreatic cancer	112
Gemcitabine	Upregulating RIP1 and RIP3	Pancreatic cancer	103
Silver nanoparticles	Upregulating RIP1 and RIP3	Pancreatic cancer	114
